# The small RNA PrrH aggravates *Pseudomonas aeruginosa*-induced acute lung injury by regulating the type III secretion system activator ExsA

**DOI:** 10.1128/spectrum.00626-23

**Published:** 2024-01-30

**Authors:** Qixuan Shi, Shenghe Zeng, Ruiqi Yu, Mo Li, Cong Shen, Xuan Zhang, Chanjing Zhao, Jianming Zeng, Bin Huang, Jieying Pu, Cha Chen

**Affiliations:** 1 Department of Laboratory Medicine, the Second Affiliated Hospital of Guangzhou University of Chinese Medicine, Guangzhou, China; 2 The Second Clinical College of Guangzhou University of Chinese Medicine, Guangzhou, China; 3 Department of Laboratory Medicine, the First Affiliated Hospital of Sun Yat-sen University, Guangzhou, China; Cinvestav-IPN, Mexico

**Keywords:** *Pseudomonas aeruginosa*, PrrH, T3SS, ExsA, lung injury

## Abstract

*Pseudomonas aeruginosa* is an opportunistic pathogen that causes acute and chronic infections in immunocompromised individuals. Small regulatory RNAs (sRNAs) regulate multiple bacterial adaptations to environmental changes, especially virulence. Our previous study showed that sRNA PrrH negatively regulates the expression of a number of virulence factors, such as pyocyanin, rhamnolipid, biofilm, and elastase in the *P. aeruginosa* strain PAO1. However, previous studies have shown that the *prrH*-deficient mutant attenuates virulence in an acute murine lung infection model. All Δ*prrH*-infected mice survived the entire 28-day course of the experiment, whereas all mice inoculated with the wild-type or the complemented mutant succumbed to lung infection within 4 days of injection, but the specific mechanism is unclear. Herein, we explored how PrrH mediates severe lung injury by regulating the expression of virulence factors. *In vivo* mouse and *in vitro* cellular assays demonstrated that PrrH enhanced the pathogenicity of PAO1, causing severe lung injury. Mechanistically, PrrH binds to the coding sequence region of the mRNA of *exsA*, which encodes the type III secretion system master regulatory protein. We further demonstrated that PrrH mediates a severe inflammatory response and exacerbates the apoptosis of A549 cells. Overall, our results revealed that PrrH positively regulates ExsA, enhances the pathogenicity of *P. aeruginosa*, and causes severe lung injury.

**IMPORTANCE:**

*Pseudomonas aeruginosa* is a Gram-negative bacterium and the leading cause of nosocomial pneumonia. The pathogenicity of *P. aeruginosa* is due to the secretion of many virulence factors. Small regulatory RNAs (sRNAs) regulate various bacterial adaptations, especially virulence. Therefore, understanding the mechanism by which sRNAs regulate virulence is necessary for understanding the pathogenicity of *P. aeruginosa* and the treatment of the related disease. In this study, we demonstrated that PrrH enhances the pathogenicity of *P. aeruginosa* by binding to the coding sequence regions of the ExsA, the master regulatory protein of type III secretion system, causing severe lung injury and exacerbating the inflammatory response and apoptosis. These findings revealed that PrrH is a crucial molecule that positively regulates ExsA. Type III-positive strains are often associated with a high mortality rate in *P. aeruginosa* infections in clinical practice. Therefore, this discovery may provide a new target for treating *P. aeruginosa* infections, especially type III-positive strains.

## INTRODUCTION

*Pseudomonas aeruginosa* is a multidrug-resistant opportunistic pathogen that causes acute and chronic infections in immunocompromised individuals with chronic obstructive pulmonary disease, cystic fibrosis, and serious burns. *P. aeruginosa* is the most common cause of nosocomial pneumonia among Gram-negative bacteria (1–6). And in recent years, *P. aeruginosa* infections cause high mortality due to its high drug resistance and virulence (7–9), which makes them extremely difficult to treat (10–14). Therefore, understanding the underlying mechanisms of *P. aeruginosa*-induced pneumonia is crucial for the treatment of the infection.

*P. aeruginosa* can adapt to the host environment due to its many virulence factors, leading to infection and disease. The virulence factors that participate in the pathogenesis of respiratory infections include the lipopolysaccharide(LPS), the type III secretion system (T3SS), pyocyanin, and rhamnolipid (15). Among the numerous virulence factors, T3SS plays a key role in disrupting the host immune system during the acute infection of *P. aeruginosa* (16–18).

T3SS is a needle-like complex of Gram-negative pathogens that includes five different components: needle structure, translocation apparatus, regulation system, effector protein, and chaperone protein (19). And the regulation of T3SS is a complex balance between different regulators (20). ExsA is the master regulator involved in the transcriptional activation of all genes of T3SS. Especially in low Ca^2+^ environments or on contact with host cells, ExsA is massively activated, activating transcription of T3SS-related genes (20, 21). Activation of T3SS rapidly disrupts host cell membranes and tight junctions between cells, undermining the innate immune response to infection (22–24). Previous studies have also confirmed that the severity of acute *P. aeruginosa* infection and high mortality rates are strongly associated with the expression of T3SS (25, 26).

The quorum-sensing system, two-component system, and small regulatory RNA (sRNA) are known virulence regulatory systems (27). Recent studies have shown that sRNAs are the primary molecules that regulate the expression of many genes and virulence factors to help pathogens adapt rapidly to the host environment (28, 29). The sRNA PrrH in *P. aeruginosa* is only encoded by two adjacent tandem genes, PrrF1 and PrrF2. Previous studies showed that PrrH is functionally homologous to the RyhB RNAs encoded by *Escherichia coli*. PrrH and RyhB RNAs are mainly involved in iron and heme metabolism (30–33). Subsequent studies showed that PrrH regulates the expression of virulence factors (31). And our previous study also showed that PrrH repressed the formation of pyocyanin, rhamnolipids, elastase, and biofilms (34). However, previous studies have shown that the *prrH-*deficient mutant attenuates the virulence in an acute lung infection model in mice. All mice infected with Δ*prrH* survived the entire 28-day course of the experiment, whereas all mice infected with the wild-type or the complemented mutant died of lung infection within 4 days of injection (31, 32), suggesting that PrrH may influence the pathogenicity through the regulation of other virulence factors. But the specific mechanism is not clear, Herein, we sought to explore the role of PrrH on bacterial-host interactions in acute lung infections.

### PrrH enhances *P. aeruginosa* cytotoxicity and causes severe lung damage

**Fig 1:**
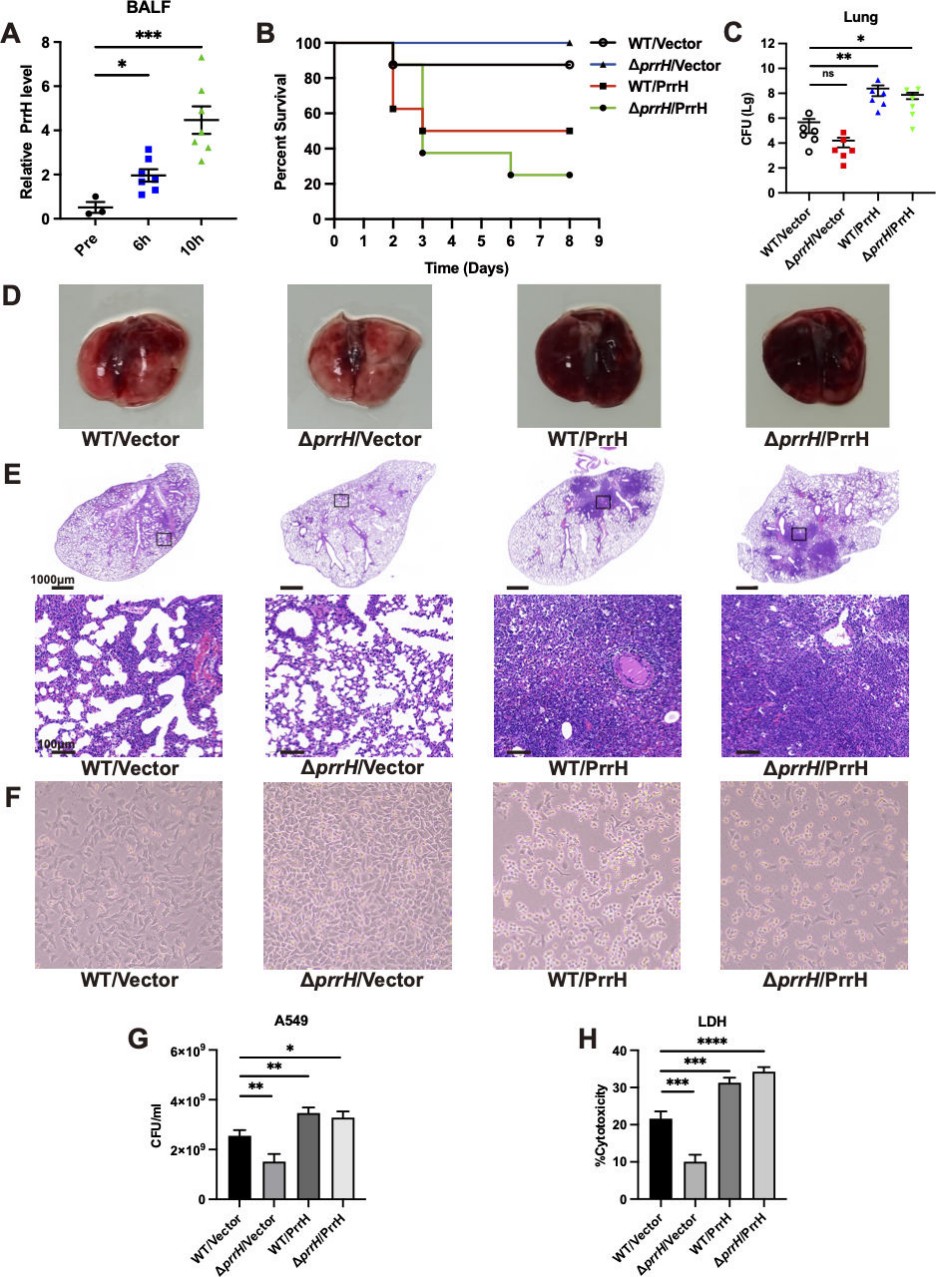
The effect of PrrH on *P. aeruginosa* pathogenicity, lung damage, and innate host immune response. (**A**) The C57BL/6 mice were infected with PAO1 strain at 1 × 10^8^ CFU, and the bacteria were extracted from alveolar lavage fluid 6 h and 10 h post-infection. PrrH expression was analyzed using qRT-PCR (Pre, uninfected group as control). (**B**) Survival curves of C57BL/6 mice infected with different *P. aeruginosa* strains (WT/Vector, Δ*prrH*/Vector, WT/PrrH, and Δ*prrH*/PrrH) at 3 × 10^7^ colony-forming units (CFU). (**C**) The C57BL/6 mice were infected with different *P. aeruginosa* strains at 3 × 10^7^ CFU for 24 h. The lung homogenate was centrifuged, and the supernatant was applied to the plate to culture and count the number of live CFU in the lung. (**D**) Lung anatomy of C57BL/6 mice infected with different *P. aeruginosa* strains at 3 × 10^7^ CFU for 24 h. (**E**) Hematoxylin and eosin staining of the lungs of C57BL/6 mice infected with different *P. aeruginosa* strains at 3 × 10^7^ CFU for 24 h. Observed under microscope, first row magnification 1.0×; second row magnification 10.0×. (**F**) Microscopic images of A549 cells infected with MOI50 of different *P. aeruginosa* strains for 12 h. (**G**) A549 cells were infected with different *P. aeruginosa* strains at MOI50 for 8 h. All bacteria in the co-culture system were recovered and counted on agar plates containing gentamicin (30 µg/mL). (**H**) A549 cells were infected with different strains at MOI50 for 12 h. The relative levels of lactate dehydrogenase in the co-culture supernatant were measured in response to the mortality of A549 cells. Values were expressed as the mean of at least three independent experiments ± SEM (*, *P* < 0.05; **, *P* < 0.01; ***, *P* < 0.001; ****, *P* < 0.0001; ns, non-significant). MOI, multiplicity of infection.

An *in vivo* acute lung infection model in which C57BL/6 mice were infected with PAO1 for 6 h and 10 h was used to investigate changes in the expression of PrrH in *P. aeruginosa* during lung infection. Bacteria from alveolar lavage fluid were collected, and PrrH was examined using quantitative real time polymerase chain reaction (qRT-PCR). The expression levels of PrrH in the 6-h and 10-h groups were 3.84-fold and 8.76-fold higher, respectively, than in the uninfected group (Fig. 1A). These results suggested that the expression of PrrH in *P. aeruginosa* increased during lung infection.

As previously described (34), the wild-type strain (WT/Vector), the *prrH* gene-deficient strain (Δ*prrH*/Vector), the *prrH*-overexpression strain (WT/PrrH), and the *prrH* complementation strain, which overexpress the *prrH* gene in the background of Δ*prrH* mutation (Δ*prrH*/PrrH), were constructed. The expression levels of the PrrH in these strains were analyzed using qRT-PCR (Fig. S1). There was no significant difference in the growth of these strains (Fig. S2). To explore the function of PrrH in *P. aeruginosa* during lung infections, these strains were intranasally inoculated into C57BL/6 mice at 3 × 10^7^colony-forming units (CFU)/mouse. The weight and physiological state of the mice were recorded daily. The result was that none of the mice infected with the Δ*prrH*/Vector strain died after 8 days, 50% of mice infected with the WT/PrrH strain were alive after 3 days, and 25% of mice infected with the Δ*prrH*/PrrH strain were alive after 6 days (Fig. 1B). The WT/PrrH strain and Δ*prrH*/PrrH strain showed higher virulence in mouse lung infections. Then, mice were further dissected at 24 h post-infection, and the bacterial load in the lungs was measured. Compared to the WT/Vector, the Δ*prrH*/Vector strain had a lower bacterial load in the lung, whereas the WT/PrrH and Δ*prrH*/PrrH strains had a higher bacterial load (Fig. 1C). After infection with the WT/Vector, the lung developed edema and hemorrhage, which were mild in the lung of mice infected with the Δ*prrH*/Vector and more severe in the lung of mice infected with the WT/PrrH and Δ*prrH*/PrrH strain (Fig. 1D). The PrrH showed more pathogenicity, consistent with previous results. To determine the effect of PrrH on the host immune response, the mice were injected with different *P. aeruginosa* strains and the lung tissues were stained with hematoxylin and eosin (H&E), and the inflammatory response was observed. The WT/PrrH-infected mice and the Δ*prrH*/PrrH-infected mice showed a higher neutrophil infiltration and inflammatory response (Fig. 1E).

Next, the virulence of the different strains infected with A549 cells was then compared, and a similar phenomenon was observed. After infection for 12 h, most of the cells infected with the WT/Vector strain maintained their normal morphology, few cells were round and refractive, changing from an adnate to a suspended state. The cells infected with the Δ*prrH*/Vector strain maintained their normal morphology and even proliferated. However, the cells infected with the WT/PrrH and Δ*prrH*/PrrH strains were crinkled, rounded, refractive, and floated in the supernatant (Fig. 1F). To quantify the mortality of A549 cells, we assayed the relative release of lactate dehydrogenase in the co-culture supernatant. Compared with the WT/Vector strain, the bacterial load and cytotoxicity were lower for the Δ*prrH*/Vector after infection of A549 cells. The bacterial load and cytotoxicity were higher in the WT/PrrH and Δ*prrH*/PrrH strains (Fig. 1G and H).

Overall, *in vivo* mouse and *in vitro* cellular experiments confirmed that PrrH knockout attenuates *P. aeruginosa* pathogenicity and growth during infection and induces a mild immune response. In contrast, overexpression of PrrH enhances bacterial pathogenicity, accelerates growth during infection, and induces a stronger inflammatory response in the host.

### PrrH promotes the expression of T3SS-related genes and targets ExsA

The primary pathogenic mechanism of *P. aeruginosa* is the secretion of various virulence factors that mediate escape from the host immune response. To investigate which virulence factors are controlled by PrrH, we examined the expression of multiple virulence factors in PrrH mutant strains. The quorum sensing (QS)-related virulence factors such as pyocyanin, elastase, rhamnolipid, and biofilm, which are negatively regulated by PrrH, were tested in a previous study (34). But the expression of these virulence factors was not compatible with PrrH-mediated cytotoxicity. Lipopolysaccharide (LPS) is a classical structural component of the outer membrane of most Gram-negative bacteria and is a potent agonist of the innate inflammatory response. We examined the expression of LPS in different PrrH mutant strains. However, LPS expression did not differ significantly among strains (Fig. S3), nor did the expression of LPS-related genes (Fig. S4).

**Fig 2:**
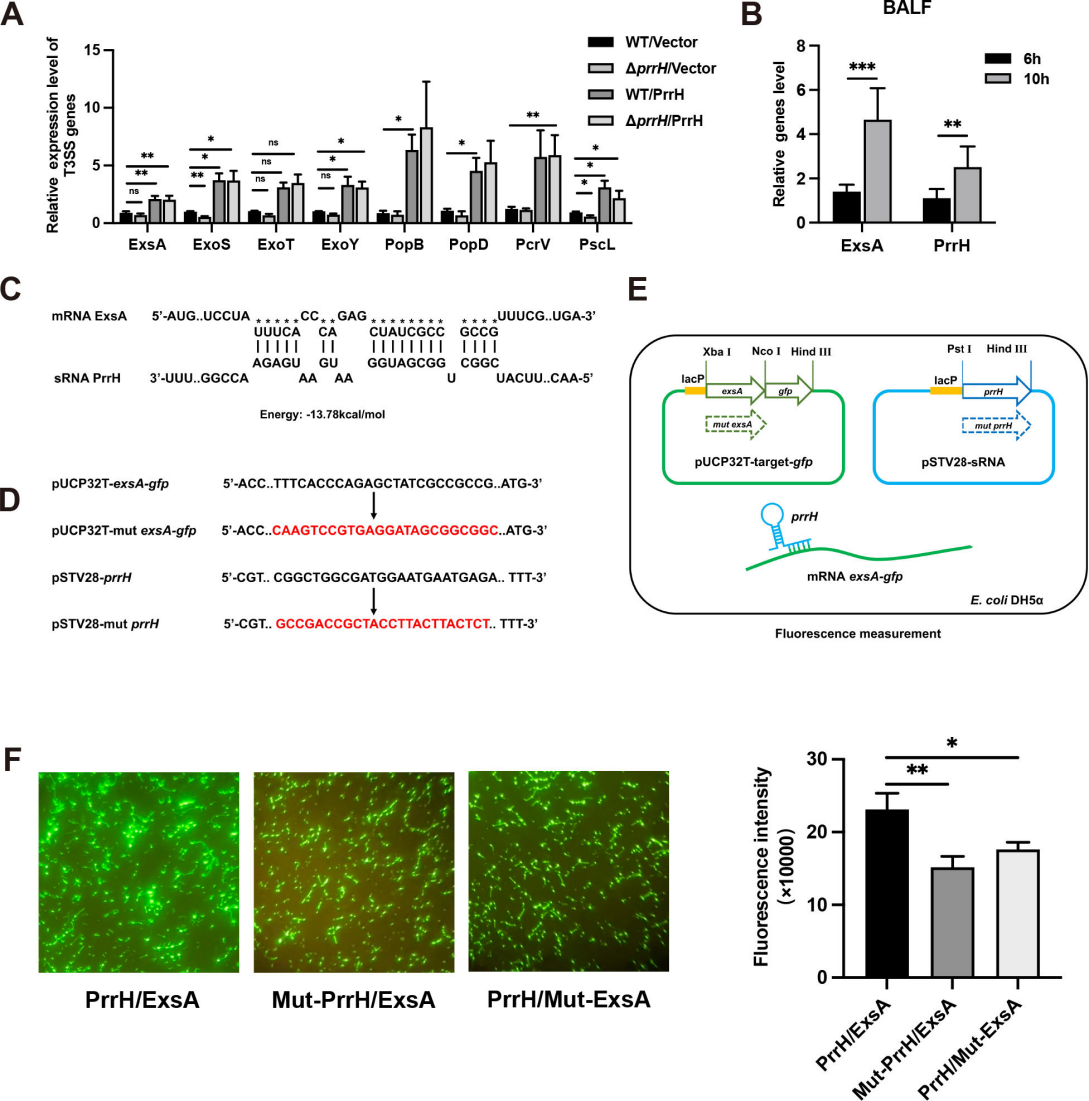
PrrH promoted the expression of T3SS-related genes and targeted ExsA, the primary T3SS regulator. (**A**) The expression of genes related to T3SS-related in different *P. aeruginosa* strains (WT/Vector, Δ*prrH*/Vector, WT/PrrH, and Δ*prrH*/PrrH). The bacteria were cultured in Luria-Bertani broth for 2 h, and the expression of the genes was detected using qRT-PCR. (**B**) The C57BL/6 mice were infected with PAO1 at 1 × 10^8^ CFU for 6 h and 8 h, bacteria were collected from the alveolar lavage fluid, and the expression of PrrH and ExsA genes was detected using qRT-PCR. (**C**) Binding sites of PrrH and ExsA as predicted by IntaRNA. (**D**) Schematic diagram of the specific site of plasmid mutation. (**E**) Principles of the *in vivo* experiment for interactions between PrrH and targets in the *E. coli* DH5α strain. (**F**) PrrH enhanced the intensity of green fluorescent protein (GFP) by binding its binding sequences at the coding sequence region of ExsA. The GFP was observed using fluorescence microscopy (left), and the intensity was measured by a Tecan Infinity M1000Pro Reader, expressed in arbitrary units as F485/535/Abs595 (right). Values were expressed as the mean of at least three independent experiments ± SEM (*, *P* < 0.05; **, *P* < 0.01; ***, *P* < 0.001; ns, non-significant).

The prediction analysis for the potential targets of PrrH based on the data in the IntaRNA database is shown in Table S1. Notably, we found that PrrH targets multiple T3SS-related genes, including the translocation apparatus, the basal body, the needle filament, the effector proteins, the regulation system, and the chaperones. We examined the expression of the representative T3SS-related genes (*exsA, exoS, exoT, exoY, popB, popD, pcrV,* and *pscL*) in different strains using qRT-PCR. The results showed that the expression of T3SS-related genes was significantly higher in the WT/PrrH and Δ*prrH*/PrrH strains compared to the WT/Vector strain (Fig. 2A). These results suggested that PrrH promotes the expression of T3SS-related genes. ExsA is a general transcriptional activator of all structural genes of the T3SS. In *in vivo* mouse experiments, the expression of both ExsA and PrrH was enhanced in *P. aeruginosa* pulmonary infection, which led us to explore the relationship between PrrH and ExsA. (Fig. 2B).

We then predicted the specific binding region of sRNA PrrH-mRNA ExsA using IntaRNA (Fig. 2C). The 24 nt sequence of PrrH predicted to bind to ExsA was located within the intergenic region of PrrH (PrrH_IG_) (Table S4). First, we constructed different plasmids: the pSTV28-PrrH plasmid (expressing PrrH), the pSTV28-mut-PrrH plasmid (expressing PrrH with reverse mutation of the predicted binding site), pUCP32T-ExsA-green fluorescent protein (GFP) plasmid (expressing ExsA fragment containing the predicted binding site), and pUCP32T-mut-ExsA-GFP plasmid (expressing ExsA fragment containing the reverse mutation of the predicted binding site) (Fig. 2D). The predicted binding sequences and mutated sequences are shown in Fig. 2D and Table S4. Then, different plasmids were introduced into *E. coli* DH5α to construct different GFP reporter systems (Fig. 2E), the unmutated strains “PrrH/ExsA” (pSTV28-PrrH, pUCP32T-ExsA-GFP), the target-mutant strains “PrrH/Mut-ExsA” (pSTV28-PrrH, pUCP32T-mut-ExsA-GFP), and the sRNA-mutant strains “Mut-PrrH/ExsA” (pSTV28-mut-PrrH, pUCP32T-ExsA-GFP). We examined the GFP fluorescence intensity values in different strains, and the results showed that the fluorescence intensity of the PrrH/ExsA strain is stronger compared to the Mut-PrrH/ExsA and the PrrH/Mut-ExsA strains, both when viewed under a microscope and when detected by an enzyme marker. This phenomenon suggests that PrrH can bind to the predicted binding site of ExsA by base complementary pairing, affecting the enhancement of GFP fluorescence intensity.

### PrrH increases the pathogenicity of *P. aeruginosa* by regulating ExsA

**Fig 3:**
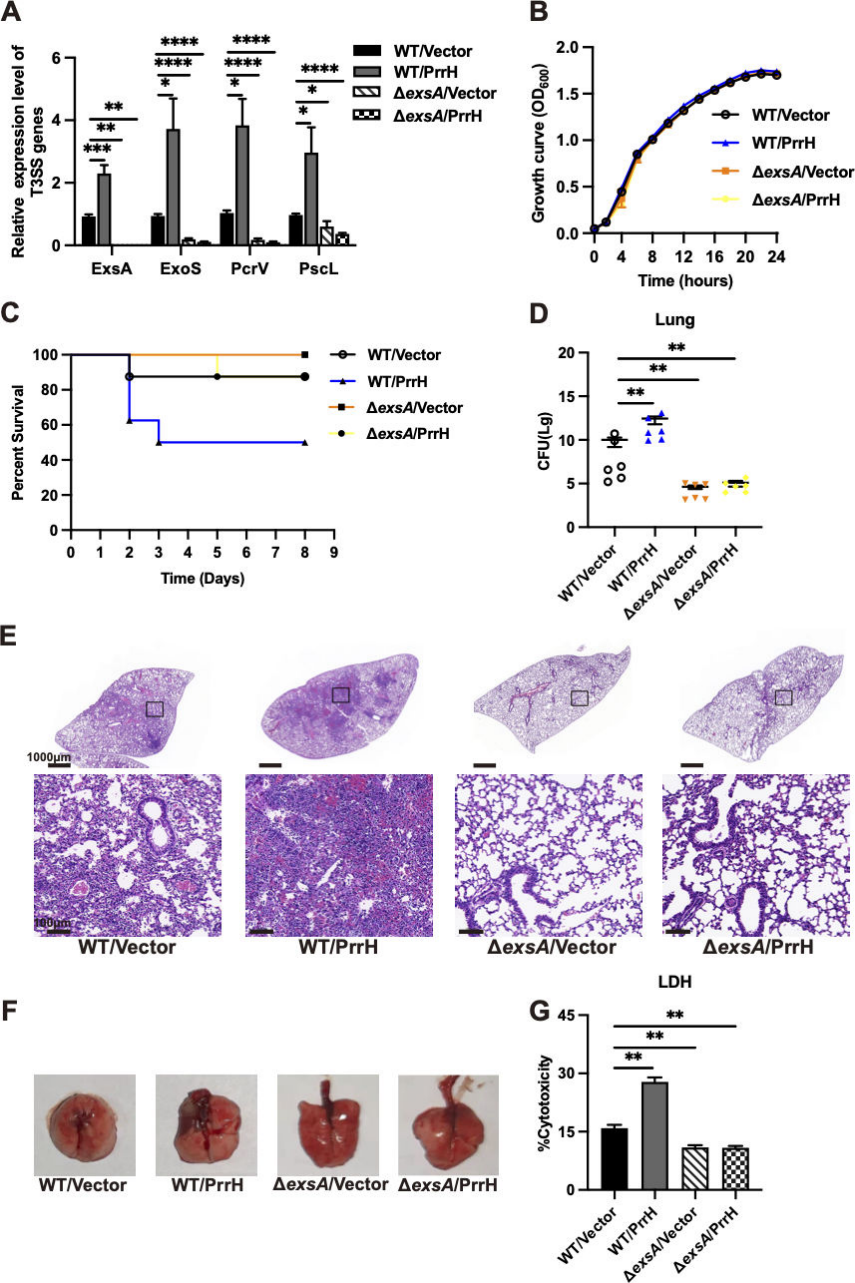
The mechanism by which PrrH increases the pathogenicity of *P. aeruginosa*. (**A**) Validation of T3SS-related gene expression in mutant strains (WT/Vector, WT/PrrH, ΔexsA/Vector, and ΔexsA/PrrH) using qRT-PCR. (**B**) Knockout of the ExsA gene in PAO1 had no effect on the growth of *P. aeruginosa*. (**C**) The C57BL/6 mice were infected with different strains at 3 × 10^7^ CFU, and the daily mortality of the mice was observed and recorded (survival rate). (**D**) The C57BL/6 mice were infected with different strains at 3 × 10^7^ CFU for 24 h. The lung homogenate was centrifuged, and the supernatant was applied to the plate to culture and count the number of live CFU in the lung. (**E**) H&E staining of the lungs of C57BL/6 mice infected with different strains at 3 × 10^7^ CFU for 24 h. (**F**) Lung anatomy of C57BL/6 mice infected with different *P. aeruginosa* strains at 3 × 10^7^ CFU for 24 h. (**G**) A549 cells were infected with different *P. aeruginosa* strains at MOI50 for 12 h and the relative levels of lactate dehydrogenase in the co-culture supernatant were measured in response to the mortality of A549 cells. The values represent the mean of at least three independent experiments ± SEM (*, *P* < 0.05; **, *P* < 0.01; ***, *P* < 0.001; ****, *P* < 0.0001). MOI, multiplicity of infection.

PrrH enhanced the pathogenicity of *P. aeruginosa*, causing severe lung damage, and promoted the expression of T3SS-related genes by targeting binding to ExsA. To explore whether PrrH regulates the cytotoxicity of *P. aeruginosa* primarily through the regulation of ExsA, an *exsA* gene-deficient strain in PAO1 (Δ*exsA*/Vector) and a *prrH*-overexpression strain in the Δ*exsA* mutation background (Δ*exsA*/PrrH) were constructed. The expression levels of T3SS-related genes in these strains were measured by qRT-PCR. The results showed that knockout of *exsA* in PAO1 significantly reduced the expression of T3SS-related genes. (Fig. 3A). And bacterial growth was not affected by knocking out the *exsA* gene. (Fig. 3B).

To evaluate the cytotoxicity of the Δ*exsA*/Vector and Δ*exsA*/PrrH strains, corresponding strains were intranasally injected into C57BL/6 mice at 3 × 10^7^ CFU/mouse. The results showed that the group of mice infected with the WT/PrrH strain had a high mortality rate. However, none of the mice infected with the Δ*exsA*/Vector strain died, and only one mouse infected with the Δ*exsA*/PrrH died on the 5th day (Fig. 3C). And the bacterial loads in the lung were low for strains with Δ*exsA*/Vector and Δ*exsA*/PrrH strains (Fig. 3D). Then, to detect the host immune response induced by the Δ*exsA*/Vector and Δ*exsA*/PrrH strains, we performed H&E staining on the lung tissue. The lung tissue sections infected with the Δ*exsA*/Vector and Δ*exsA*/PrrH strains were pale, implying low neutrophil infiltration and weak inflammatory response (Fig. 3E). After dissecting the mice, we found lung tissue edema and hemorrhage in the lungs were slighter for mice infected with Δ*exsA*/Vector and Δ*exsA*/PrrH strains (Fig. 3F). These results suggested that knockout of *exsA* significantly reduced the pathogenicity of *P. aeruginosa*, and overexpression of *prrH* in the *exsA* mutant background did not restore the virulence of the strain. A similar phenomenon was observed in A549 cells, where the Δ*exsA*/Vector and Δ*exsA*/PrrH strains showed weak cytotoxicity (Fig. 3G).

*Consequentlly, in vivo* mouse and *in vitro* cellular experiments confirmed that knocking out *exsA* significantly reduced the cytotoxicity of *P. aeruginosa*, and the overexpression of PrrH in the Δ*exsA* did not also restore the virulence of the strain. This suggests that PrrH enhanced the cytotoxicity of *P. aeruginosa* primarily through the regulation of ExsA.

### PrrH promotes host inflammatory response and apoptosis by regulating ExsA

**Fig 4:**
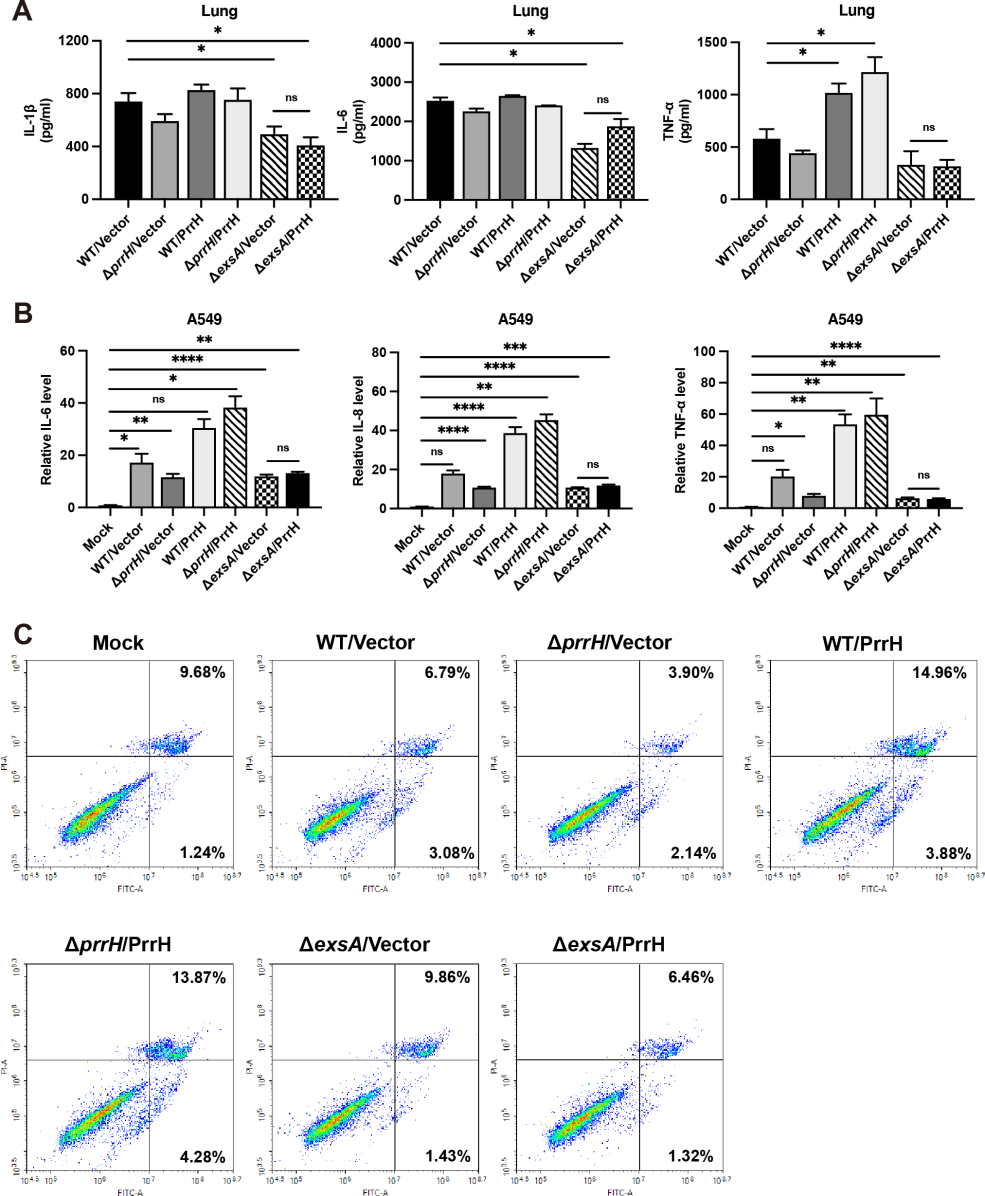
PrrH promotes host inflammatory response and apoptosis . (**A**)The effect of different *P. aeruginosa* strains on inflammation. C57BL/6 mice were infected with different *P. aeruginosa* strains at 3 × 10^7^CFU. The expression of inflammatory factors (IL-1β, IL-6, and TNF-α) in the lungs was detected using the ELISA kit (compared with WT/Vector group). (**B**)A549 cells infected with MOI50 of different *P. aeruginosa* strains for 4 h. The expression of inflammatory factors (IL-8, IL-6, and TNF-α) was detected using qRT-PCR (compared with the uninfected group). (**C**)A549 cells infected with different *P. aeruginosa* strains for 8 h. The apoptosis was detected using flow cytometry. Values were expressed as the mean of at least three independent experiments ± SEM (*, *P* < 0.05; **, *P* < 0.01; ***, *P* < 0.001; ****, *P* < 0.0001; ns, non-significant). MOI, multiplicity of infection.

PrrH exacerbated the lung injury and the inflammatory response of the host. Accordingly, we measured the expression levels of several inflammatory cytokines in mouse lung homogenates to assess the host inflammatory response caused by PrrH. Compared with WT/Vector strain, infection with the WT/PrrH and the Δ*prrH*/PrrH strains increased the expression of tumor necrosis factor-α (TNF-α) and interleukin-1β (IL-1β). However, infection with the Δ*prrH*/Vector, Δ*exsA*/Vector, and the Δ*exsA*/PrrH strains suppressed the expression of TNF-α and IL-1β (Fig. 4A). There was no significant difference in inflammatory factor interleukin-6 (IL-6) levels between the strains except for the Δ*exsA*/Vector and Δ*exsA*/PrrH strains. *In vivo* results revealed that PrrH induced inflammation in the host by primarily upregulating the secretion of TNF-α and IL-1β. Infection with the Δ*exsA*/Vector strains resulted in reduced expression of inflammatory factors, and infection with the Δ*exsA*/PrrH strains did not increase inflammatory factor expression, suggesting that PrrH-mediated inflammation is indeed mediated by modulation of ExsA. We also examined the expression of inflammatory factors in the A549 cells. Compared to the uninfected group, the cells infected with the WT/PrrH and the Δ*prrH*/PrrH strains increased the expression of TNF-α expressed by 50-fold, IL-6 by 30-fold, and IL-8 by 40-fold. The cells infected with the Δ*prrH*/Vector, Δ*exsA*/Vector, and Δ*exsA*/PrrH strains increased the expression of TNF, IL-6, and IL-8 by about 10-fold (Fig. 4B). Similarly, infection with the Δ*exsA*/PrrH strains did not increase the expression of inflammatory factors, a phenomenon consistent with that in mice. To investigate the mechanism of PrrH-mediated cell death, we examined the apoptosis rate of A549 cells infected with different *P. aeruginosa* strains. Compared with the WT group (9.87%), the apoptosis rate (6.04%) was lower in A549 cells infected with Δ*prrH*/Vector strain. However, the apoptosis rate was higher in A549 cells infected with WT/PrrH and Δ*prrH*/PrrH strains (18.84%; 18.15%). The apoptosis rates were lower for A549 cells infected with Δ*exsA*/Vector and Δ*exsA*/PrrH strains (11.29%; 7.78%) (Fig. 4C). Generally, PrrH enhanced the host inflammatory response and exacerbated apoptosis in A549 cells.

Overall, these results show that PrrH induces severe lung damage, inflammation, and apoptosis by positively regulating ExsA expression.

## DISCUSSION

*P. aeruginosa* is the leading Gram-negative cause of nosocomial pneumonia (1–6). *P. aeruginosa* relies primarily on the secretion of large amounts of virulence factors to evade host immunity and successfully infect and cause disease (15, 35). In this study, we found that the expression of PrrH is increased during acute lung infection and that it causes severe lung injury and hemorrhage. Furthermore, we found that PrrH enhanced *P. aeruginosa*-induced lung injury mainly through the regulation of ExsA, a master regulator of T3SS. In addition, PrrH enhanced the host inflammatory response and the apoptosis of epithelial cell. Thus, this study illustrated how PrrH helps to regulate bacterial pathogenicity and facilitates escape in *P. aeruginosa* lung infections.

Numerous previous studies have implicated sRNAs in the regulation of *P. aeruginosa* pathogenicity (21, 36–38). PrrH was originally described as a “heme-responsive RNA” (31). Our previous study showed that PrrH repressed the formation of QS system-related virulence including pyocyanin, rhamnolipids, elastase, and biofilms (34). However, in an acute mice lung infection model, previous studies have shown that the *prrH*-deficient mutant attenuates virulence, but the specific mechanism is not clear (31, 32). In this study, we found that the strains overexpressing PrrH were highly cytotoxic and caused a significant injury and hemorrhage in lung. Therefore, we speculated that PrrH may influence pathogenicity by regulating other virulence factors. *P. aeruginosa* can adapt to the adverse host and contribute to successful infection by secreting a variety of virulence factors, including LPS, T3SS, T6SS, and biofilms (15, 35). The LPS is the endotoxin and inflammatory activator in Gram-negative bacteria (39). We examined LPS expression in several PrrH mutant strains and found that PrrH has no effect on both LPS protein synthesis and LPS-related gene expression (Fig. S3 and S4). We then analyzed the possible target genes of PrrH by IntaRNA prediction and found that it targets several genes related to the T3SS. Therefore, we further validated the relationship of PrrH and T3SS.

T3SS is a complex bacterial machinery consisting of five distinct components: needle structure; translocation apparatus; regulatory system; chaperones and effectors (19). In previous studies, the expression of T3SS had been proven that it can be directly regulated by multiple regulators (e.g., expressor PsrA and repressor PtrA/AtrR) and other relative material which participate in regulating the T3SS (e.g., PsrA/RpoS cAMP/Vfr, GacSA-RsmYZ-RsmA system, and VqsM, RetS, LadS, MvaT, and MvaU) (19). The IntaRNA prediction results showed that PrrH targets several structural T3SS-related genes (Table S1). We further selected representative genes from the five components and examined the expression of these genes in mutant strains of PrrH (Fig. 2A). The results showed that the expression of all T3SS-related genes was significantly higher in the *prrH*-overexpressing strains. One of them, ExsA, is a universal T3SS regulator. It is involved in transcriptionally activating the five operons encoding all structural and the five regulatory genes (40, 41). Therefore, our hypothesis is that PrrH may activate the T3SS through regulation of the transcription activator ExsA. The GFP fluorescent reporter assays proved that PrrH does indeed bind to ExsA mRNA. Based on the IntaRNA prediction, it is possible that PrrH also targets other T3SS-related genes. The specific regulatory relationships of PrrH and related genes can be subsequently verified by qRT-PCR and the GFP fluorescent reporter systems.

The results show that compared to the PrrH/Mut-ExsA strains and the Mut-PrrH/ExsA strains, the fluorescence intensity of GFP significantly enhanced in PrrH/ExsA strain (Fig. 2F). As our guess, when PrrH binds to the predicted sequence of ExsA, it may open the hairpin structure and promotes translation of GFP, resulting in bright fluorescence. The results also show that the PrrH_IG_ sequence is essential for binding and potentially activating ExsA translation.

T3SS is an important virulence factor in the acute phase of *P. aeruginosa* infection. It can activate multiple host immune pathways. The effector ExoT activates the mitochondrial/cytochrome c-dependent apoptotic pathway and effectively blocks necrotic cell death in Hela cells (17). The formation of a translocation competent T3SS is essential for the triggering of caspase-1 activation and IL-1β maturation (18). The effectors ExoS and ExoT regulated the expression of genes associated with acute and chronic infections in an nuclear factor-kappa B (NF-κB)-dependent manner (42). In our study, we found that PrrH significantly enhanced the host inflammatory response (Fig. 2E), and we demonstrated that the upregulation of inflammatory factor expression by PrrH and the increased rate of apoptosis were achieved mainly by regulating ExsA (Fig. 4A and B). These findings are consistent with the previously reported T3SS-mediated immune response of the host.

An *in vivo* acute lung infection model demonstrated that the expression of PrrH in *P. aeruginosa* increased during lung infection (Fig. 1A). It has previously been reported that *prrH* is an iron ion and heme-responsive sRNA (30, 31). The ferric uptake regulator can bind to the promoter region of *prrH* to regulate *prrH* expression (43). Therefore, we hypothesized that during acute lung infection, hemorrhage from lung injury releases large amounts of heme from iron ions, which affects *prrH* expression. Thus, PrrH is a sensor that rapidly responds to environmental changes, activating downstream virulence factors to enhance virulence and help *P. aeruginosa* escape.

In summary, our study revealed that the expression of PrrH is elevated during *P. aeruginosa* lung infection, which mediates severe lung injury by regulating the T3SS activator ExsA. This function of PrrH may provide a potential target for the clinical treatment of *P. aeruginosa* infections, especially T3SS-positive bacteria.

## MATERIALS AND METHODS

### Bacterial strains and culture conditions

The PAO1 strain was donated by Professor Zhou (Children’s Hospital of Chongqing Medical University). The strains were cultured in fresh Luria-Bertani (LB) medium or LB plates (1.5% agar) at 37°C. If needed, antibiotics were added to the medium or plates at the following concentrations: 30 µg/mL gentamicin and 16 µg/mL chloramphenicol. The construction of PAO1 *prrH*-deficient mutants was performed in our previous study. Details of the strains are provided in Table S2.

### Construction of the *exsA*-deficient mutants in PAO1

The sacB-based suicide vector system was used for the knockout of *exsA* in PAO1, as described in our previous study (34). Briefly, the upstream and downstream sequences of the *exsA,* termed flanking fragment A and flanking fragment B, were amplified by fusion PCR and ligated to form a recombinant fragment AB. The AB fragment was cloned into *XbaI/SacI* sites in pGSM to generate the recombinant plasmid pGSM-Δ*exsA* and was then transformed into PAO1 to generate *exsA* mutant strain (Δ*exsA*). An empty plasmid (pROp200) and an overexpression plasmid (pROp200-*prrH*) were transformed into Δ*exsA* to generate *exsA* mutant strains with empty plasmids (Δ*exsA*/Vector) and *exsA* mutant strains with a *prrH*-overexpression plasmid (Δ*exsA*/PrrH), respectively.

### Cell culture and infection

Human pulmonary epithelial cell line A549 (ATCC: CCL-185) cells were cultured in Dulbecco's Modification of Eagle's Medium (DMEM) (Gibco, Carlsbad, CA) containing 10% Fetal Bovine Serum (FBS) (Gibco, Carlsbad, CA) at 37°C under 5% CO_2_. Cells were passaged at a ratio of 1:5 each time and cultured continuously for 3 days. A549 cells were seeded in well plates and incubated at 37°C for 14 h. The indicated strains underwent a logarithmic growth phase and were collected from the solution and resuspended in phosphate buffered solution (PBS). Prior to infection, the original cell culture medium was removed and replaced with DMEM containing 1% FBS. Cells were infected with bacteria at a specific dose based on the absorbance of the suspensions at 600 nm (OD_600_). Bacterial CFU and OD_600_ values were obtained using the formula 22.031 × OD_600_ + 0.8278 = CFU (unit: 10^8^ CFU/mL).

### Mouse infections

C57BL/6 mice (male, 6 weeks old, weighing 17 g–19 g) were purchased from Guangdong Animal Centre. The indicated strains, which underwent logarithmic growth phases were removed from the solution and resuspended in PBS. Appropriate dilutions were prepared according to the OD_600_. Mice were anesthetized with isoflurane (4%) and intranasally inoculated with 30µL of bacterial solution. Mice were infected at a dose of 1 × 10^8^CFU and divided into two infection groups (seven mice per group) according to different infection times (6h and 10h). The PAO1 bacteria in the alveolar lavage fluid of the mice were collected and tested simultaneously with uninfected strains for PrrH gene expression. The infectious dose used in the survival curve was 3 × 10^7^CFU (lethal dose 50%, LD50) per mouse (eight mice for each bacterial strain). Body weight and mortality of the mice were observed and recorded daily. Survival curves were plotted based on mortality in each group within 7 days of infection. Mice were infected with a dose of 3 × 10^7^CFU and euthanized after 24 h. Lung tissues and lungs were homogenized for gene expression and CFU count assays. H&E staining of mouse lung tissues was performed to observe inflammatory infiltration in the lungs. Briefly, lung tissues were fixed in 4% paraformaldehyde (Biosharp, China) overnight, dehydrated in ethanol, embedded in paraffin, sectioned, and stained with hematoxylin and eosin.

### Colony-Forming Units

*For in vivo* mice experiments, lung tissues were ground into lung homogenates, and supernatants were collected after centrifugation at 1,000 rpm and then diluted with PBS. Then, 10 µL of the diluted solution was titrated onto a gentamicin-resistant plate (30 µg/mL) and incubated at 37°C for 16 h. The number of monoclonal clones on the plate was counted and converted into the original bacterial load of the lung homogenate with a dilution multiple. For *in vitro* cellular experiments, A549 cells were seeded on a six-well plate (8 × 10^5^/well) for 1 day and then infected with the indicated strains at a multiplicity of infection (MOI) of 50 for 8 h. The supernatant from the co-culture system was removed and 2 mL of 0.1% Triton X-100 was added to the well plate to completely lyse the cells and release the bacteria. The bacteria in the supernatant and the released bacteria were collected and diluted with PBS. Fifty microliters of the diluted bacterial solution was applied to a gentamicin-resistant plate (30 µg/mL) and incubated at 37°C for 16 h. The number of monoclonal clones was counted and converted to the bacterial load in a co-culture system based on a dilution multiple.

### LPS assay

A centrifuge can be used at 13,000 rpm at room temperature to harvest 2 mL–5 mL of bacterial cells. LPS is extracted from concentrated bacteria by kit (iNtRON Biotechnology), and added to 30 μL–50 μL of 10mM Tris-HCl buffer to fully dissolve it. Then the LPS concentration was measured by the LPS ELISA kit.

### Cytotoxicity assay

In principle, cytotoxicity assays are based on the quantitative analysis of cytotoxicity by measuring the activity of lactate dehydrogenase (LDH) released into the culture medium. Briefly, A549 cells were seeded on a 96-well plate (5 × 10^4^/well) 1 day before infection and infected with the indicated bacterial strains at an MOI of 50 at 37°C with 5% CO_2_ for 12 h. Three control groups were established: cell-free culture wells (DMEM control), uninfected cell wells (cell control), and uninfected cell wells for maximum lysis (positive control). The LDH release reagent was added to the maximum lysis well for 1 h and the sample was collected according to the manufacturers’ instructions for the LDH cytotoxicity assay kit (Beyotime, Shanghai, China). The absorbance values at 490 nm (OD_490_) and 900 nm (OD_900_) were recorded simultaneously, and OD_900_ was subtracted from OD_490_. The percentage of cytotoxicity was calculated using the following formula: (A_co-culture_ − A_cell control_) / (A_positive control_ − A_cell control_) × 100%.

### GFP reporter assays

The GFP reporter plasmid system (pUCP32T-gfp) was used for *exsA* according to a previously described method. The putative binding sites of PrrH targeting ExsA were predicted by IntaRNA. A wild-type fragment of ExsA mRNA containing putative binding sites for PrrH was amplified through PCR and inserted into the XbaI/NcoI sites upstream of the first codon of GFP in the pUCP32T-gfp plasmid to generate a translational fusion plasmid, pUCP32T-*exsA-gfp*. Similarly, a mutated mut-exsA fragment was inserted into the XbaI/NcoI site upstream of the first codon of GFP in the pUCP32T-gfp plasmid to generate a mutant plasmid pUCP32T-*mut exsA-gfp*. The pUCP32T-*exsA-gfp* or pUCP32T-*mut exsA-gfp* was then transformed into competent *E. coli* DH5α cells with pSTV28-*prrH* or pSTV28-*mut PrrH*. Three DH5α strains (PrrH/ExsA, PrrH/Mut-ExsA, Mut-PrrH/ExsA) were collected through centrifugation and resuspended in PBS. Absorbance (Abs600) and fluorescence intensity (F485/535) were measured using a BioTek Synergy H1 microplate reader (BioTek, Winooski, VT). The GFP activity was expressed in arbitrary units as F485/535/Abs600. In addition, 10 µL of the bacterial solution was pipetted onto the slide, and the fluorescence was observed using a Nikon ECLIPSE Ti2-U fluorescence microscope (Nikon, Tokyo, Japan).

### Real-time PCR

Bacteria and cells were collected through centrifugation and lysed with the TRIzol Reagent (Takara Bio Inc.). Total RNA was extracted according to the kit instructions and quantified using a Nanotrap 2000 spectrophotometer. The RNA (1 µg) was reverse transcribed using the PrimeScript RT reagent kit (TaKaRa, Dalian, China). The cDNA was subjected to qPCR on a ViiATM 7 Dx system (Applied Biosystems, Foster, CA, USA) using SYBR green Premix Pro Taq HS qPCR Kit (Accurate Biology, Changsha, China). The expression levels of the target genes were normalized to the expression of the reference genes (*rpoD* or *β-actin*) and analyzed using the relative threshold cycling (2^−ΔΔCt^) method. The primer sequences used are listed in Table S3.

### Cytokine assay

The production of cytokines in lung homogenates or cell supernatants was measured using an ELISA kit (MULTI SCIENCES, for IL-6 and TNF-α; Sangon Biotech, for IL-1β) according to the manufacturer’s instructions.

### Flow cytometry for apoptosis

A549 cells inoculated into 12-well plates (4 × 10^5^/well) were infected with the indicated strain at an MOI of 50 for 8 h. Cells in the well plates were then digested with trypsin (0.25%) and collected by centrifugation. The collected cells were resuspended with 500 µL binding buffer according to the instructions of the Apoptosis Detection Kit (KeyGEN BioTECH, Jiangsu, China) and stained with 5 µL membrane-bound protein (Annexin V-FITC) and 5 µL propidium iodide for 15 min at room temperature. The stained cells were analyzed by flow cytometry (Novo Quanteon) within 1 h .

### Statistical analysis

At least three independent experiments were performed for each separate set of assays. Data are presented as mean ± SEM using GraphPad Prism 9 (GraphPad Software, San Diego, CA). Statistical significance was determined by *t*-test between two groups, with *P*-values represented as *P, 0.05, **P, 0.01, and ***P, 0.001.

## SUPPLEMENTAL MATERIAL

Supplemental material spectrum.00626-23-s0001.pdf Fig. S1 to S4; Tables S1 to S4.

ASM does not own the copyrights to Supplemental Material that may be linked to, or accessed through, an article. The authors have granted ASM a non-exclusive, world-wide license to publish the Supplemental Material files. Please contact the corresponding author directly for reuse.

## References

[1] Luyt CE, Hékimian G, Koulenti D, Chastre J. 2018. Microbial cause of ICU-acquired pneumonia: hospital-acquired pneumonia versus ventilator-associated pneumonia. Curr Opin Crit Care 24:332–338. doi:10.1097/MCC.0000000000000526 30036192

[2] Jang JH, Yeo HJ, Kim T, Cho WH, Min KH, Hong S-B, Baek A-R, Lee H-K, Kim C, Chang Y, Park HK, Oh JY, Lee HB, Bae S, Moon JY, Yoo KH, Gil H-I, Jeon K. 2022. Microbiologic pattern and clinical outcome of non-ICU-acquired pneumonia: Korean HAP registry analysis. Korean J Intern Med 37:800–810. doi:10.3904/kjim.2021.348 35811368 PMC9271727

[3] Naidus EL, Lasalvia MT, Marcantonio ER, Herzig SJ. 2018. The diagnostic yield of noninvasive microbiologic sputum sampling in a cohort of patients with clinically diagnosed hospital-acquired pneumonia. J Hosp Med 13:34–37. doi:10.12788/jhm.2868 29073317 PMC6239197

[4] Sano M, Shindo Y, Takahashi K, Okumura J, Sakakibara T, Murakami Y, Iguchi M, Yagi T, Matsui S, Hasegawa Y. 2022. Risk factors for antibiotic resistance in hospital-acquired and ventilator-associated pneumonia. J Infect Chemother 28:745–752. doi:10.1016/j.jiac.2022.02.012 35219577

[5] Yin Y, Zhao C, Li H, Jin L, Wang Q, Wang R, Zhang Y, Zhang J, Wang H. 2021. Clinical and microbiological characteristics of adults with hospital-acquired pneumonia: a 10-year prospective observational study in China. Eur J Clin Microbiol Infect Dis 40:683–690. doi:10.1007/s10096-020-04046-9 33029764 PMC7540435

[6] Feng D-Y, Zhou Y-Q, Zou X-L, Zhou M, Wu W-B, Chen X-X, Wang Y-H, Zhang T-T. 2019. Factors influencing mortality in hospital-acquired pneumonia caused by gram-negative bacteria in China. J Infect Public Health 12:630–633. doi:10.1016/j.jiph.2019.02.014 30824328

[7] Micek ST, Wunderink RG, Kollef MH, Chen C, Rello J, Chastre J, Antonelli M, Welte T, Clair B, Ostermann H, Calbo E, Torres A, Menichetti F, Schramm GE, Menon V. 2015. An international multicenter retrospective study of Pseudomonas aeruginosa nosocomial pneumonia: impact of multidrug resistance. Crit Care 19:219. doi:10.1186/s13054-015-0926-5 25944081 PMC4446947

[8] Borgatta B, Gattarello S, Mazo CA, Imbiscuso AT, Larrosa MN, Lujàn M, Rello J. 2017. The clinical significance of pneumonia in patients with respiratory specimens harbouring multidrug-resistant Pseudomonas aeruginosa: a 5-year retrospective study following 5667 patients in four general ICUs. Eur J Clin Microbiol Infect Dis 36:2155–2163. doi:10.1007/s10096-017-3039-z 28624864

[9] Poovieng J, Sakboonyarat B, Nasomsong W. 2022. Bacterial etiology and mortality rate in community-acquired pneumonia, healthcare-associated pneumonia and hospital-acquired pneumonia in Thai university hospital. Sci Rep 12:9004. doi:10.1038/s41598-022-12904-z 35637232 PMC9150030

[10] Djordjevic ZM, Folic MM, Jankovic SM. 2017. Distribution and antibiotic susceptibility of pathogens isolated from adults with hospital-acquired and ventilator-associated pneumonia in intensive care unit. J Infect Public Health 10:740–744. doi:10.1016/j.jiph.2016.11.016 28189513

[11] Meschiari M, Orlando G, Kaleci S, Bianco V, Sarti M, Venturelli C, Mussini C. 2021. Combined resistance to ceftolozane-tazobactam and ceftazidime-avibactam in extensively drug-resistant (XDR) and multidrug-resistant (MDR) Pseudomonas aeruginosa: resistance predictors and impact on clinical outcomes besides implications for antimicrobial stewardship programs. Antibiotics (Basel) 10:1224. doi:10.3390/antibiotics10101224 34680805 PMC8532599

[12] Miyoshi-Akiyama T, Tada T, Ohmagari N, Viet Hung N, Tharavichitkul P, Pokhrel BM, Gniadkowski M, Shimojima M, Kirikae T. 2017. Emergence and spread of epidemic multidrug-resistant Pseudomonas aeruginosa. Genome Biol Evol 9:3238–3245. doi:10.1093/gbe/evx243 29202180 PMC5726472

[13] Kunz Coyne AJ, El Ghali A, Holger D, Rebold N, Rybak MJ. 2022. Therapeutic strategies for emerging multidrug-resistant Pseudomonas aeruginosa. Infect Dis Ther 11:661–682. doi:10.1007/s40121-022-00591-2 35150435 PMC8960490

[14] Mahto M, Shah A, Show KL, Moses FL, Stewart AG. 2021. Pseudomonas aeruginosa in Nepali hospitals: poor outcomes amid 10 years of increasing antimicrobial resistance. Public Health Action 11:58–63. doi:10.5588/pha.21.0048 PMC8575381 34778017

[15] Qin S, Xiao W, Zhou C, Pu Q, Deng X, Lan L, Liang H, Song X, Wu M. 2022. Pseudomonas aeruginosa: pathogenesis, virulence factors, antibiotic resistance, interaction with host, technology advances and emerging therapeutics. Signal Transduct Target Ther 7:199. doi:10.1038/s41392-022-01056-1 35752612 PMC9233671

[16] Faure E, Mear JB, Faure K, Normand S, Couturier-Maillard A, Grandjean T, Balloy V, Ryffel B, Dessein R, Chignard M, Uyttenhove C, Guery B, Gosset P, Chamaillard M, Kipnis E. 2014. Pseudomonas aeruginosa type-3 secretion system dampens host defense by exploiting the NLRC4-coupled inflammasome. Am J Respir Crit Care Med 189:799–811. doi:10.1164/rccm.201307-1358OC 24555512

[17] Shafikhani SH, Morales C, Engel J. 2008. The Pseudomonas aeruginosa type III secreted toxin ExoT is necessary and sufficient to induce apoptosis in epithelial cells. Cell Microbiol 10:994–1007. doi:10.1111/j.1462-5822.2007.01102.x 18053004 PMC10952005

[18] Galle M, Schotte P, Haegman M, Wullaert A, Yang HJ, Jin S, Beyaert R. 2008. The Pseudomonas aeruginosa type III secretion system plays a dual role in the regulation of caspase-1 mediated IL-1beta maturation. J Cell Mol Med 12:1767–1776. doi:10.1111/j.1582-4934.2007.00190.x 18081695 PMC3918092

[19] Horna G, Ruiz J. 2021. Type 3 secretion system of Pseudomonas aeruginosa. Microbiol Res 246:126719. doi:10.1016/j.micres.2021.126719 33582609

[20] Galle M, Carpentier I, Beyaert R. 2012. Structure and function of the type III secretion system of Pseudomonas aeruginosa. Curr Protein Pept Sci 13:831–842. doi:10.2174/138920312804871210 23305368 PMC3706959

[21] Janssen KH, Corley JM, Djapgne L, Cribbs JT, Voelker D, Slusher Z, Nordell R, Regulski EE, Kazmierczak BI, McMackin EW, Yahr TL. 2020. Hfq and sRNA 179 inhibit expression of the Pseudomonas aeruginosa cAMP-Vfr and type III secretion regulons. mBio 11:e00363-20. doi:10.1128/mBio.00363-20 32546612 PMC7298702

[22] Grandjean T, Boucher A, Thepaut M, Monlezun L, Guery B, Faudry E, Kipnis E, Dessein R. 2017. The human NAIP-NLRC4-inflammasome senses the Pseudomonas aeruginosa T3SS inner-rod protein. Int Immunol 29:377–384. doi:10.1093/intimm/dxx047 28992059

[23] Elsen S, Huber P, Bouillot S, Couté Y, Fournier P, Dubois Y, Timsit JF, Maurin M, Attrée I. 2014. A type III secretion negative clinical strain of Pseudomonas aeruginosa employs a two-partner secreted exolysin to induce hemorrhagic pneumonia. Cell Host Microbe 15:164–176. doi:10.1016/j.chom.2014.01.003 24528863

[24] Moir DT, Bowlin NO, Berube BJ, Yabut J, Mills DM, Nguyen GT, Aron ZD, Williams JD, Mecsas J, Hauser AR, Bowlin TL. 2020. A structure-function-inhibition analysis of the Pseudomonas aeruginosa type III secretion needle protein PscF. J Bacteriol 202:e00055-20. doi:10.1128/JB.00055-20 32601072 PMC7925083

[25] Sarges E do S, Rodrigues YC, Furlaneto IP, de Melo MVH, Brabo G da C, Lopes KCM, Quaresma A, Lima L, Lima KVB. 2020. Pseudomonas aeruginosa type III secretion system virulotypes and their association with clinical features of cystic fibrosis patients. Infect Drug Resist 13:3771–3781. doi:10.2147/IDR.S273759 33116695 PMC7588269

[26] Zhang J, Chu Y, Wang P, Ji X, Li X, Wang C, Peng Y. 2014. Clinical outcomes of multidrug resistant Pseudomonas aeruginosa infection and the relationship with type III secretion system in patients with diabetic foot. Int J Low Extrem Wounds 13:205–210. doi:10.1177/1534734614545878 25106442

[27] Balasubramanian D, Schneper L, Kumari H, Mathee K. 2013. A dynamic and intricate regulatory network determines Pseudomonas aeruginosa virulence. Nucleic Acids Res 41:1–20. doi:10.1093/nar/gks1039 23143271 PMC3592444

[28] Sonnleitner E, Romeo A, Bläsi U. 2012. Small regulatory RNAs in Pseudomonas aeruginosa. RNA Biol 9:364–371. doi:10.4161/rna.19231 22336763

[29] Liu P, Yue C, Liu L, Gao C, Lyu Y, Deng S, Tian H, Jia X. 2022. The function of small RNA in Pseudomonas aeruginosa. PeerJ 10:e13738. doi:10.7717/peerj.13738 35891650 PMC9308961

[30] Oglesby-Sherrouse AG, Vasil ML. 2010. Characterization of a heme-regulated non-coding RNA encoded by the prrF locus of Pseudomonas aeruginosa. PLoS One 5:e9930. doi:10.1371/journal.pone.0009930 20386693 PMC2851614

[31] Reinhart AA, Powell DA, Nguyen AT, O’Neill M, Djapgne L, Wilks A, Ernst RK, Oglesby-Sherrouse AG. 2015. The prrF-encoded small regulatory RNAs are required for iron homeostasis and virulence of Pseudomonas aeruginosa. Infect Immun 83:863–875. doi:10.1128/IAI.02707-14 25510881 PMC4333466

[32] Reinhart AA, Nguyen AT, Brewer LK, Bevere J, Jones JW, Kane MA, Damron FH, Barbier M, Oglesby-Sherrouse AG, McCormick B. 2017. The Pseudomonas aeruginosa PrrF small RNAs regulate iron homeostasis during acute murine lung infection. Infect Immun 85:e00764-16. doi:10.1128/IAI.00764-16 28289146 PMC5400841

[33] Wilson T, Mouriño S, Wilks A. 2021. The heme-binding protein PhuS transcriptionally regulates the Pseudomonas aeruginosa tandem sRNA prrF1,F2 locus. J Biol Chem 296:100275. doi:10.1016/j.jbc.2021.100275 33428928 PMC7948967

[34] Lu Y, Li H, Pu J, Xiao Q, Zhao C, Cai Y, Liu Y, Wang L, Li Y, Huang B, Zeng J, Chen C. 2019. Identification of a novel RhlI/R-PrrH-LasI/Phzc/PhzD signalling cascade and its implication in P. aeruginosa virulence. Emerg Microbes Infect 8:1658–1667. doi:10.1080/22221751.2019.1687262 31718472 PMC6853234

[35] Jurado-Martín I, Sainz-Mejías M, McClean S. 2021. Pseudomonas aeruginosa: an audacious pathogen with an adaptable arsenal of virulence factors. Int J Mol Sci 22:3128. doi:10.3390/ijms22063128 33803907 PMC8003266

[36] Lu P, Wang Y, Zhang Y, Hu Y, Thompson KM, Chen S. 2016. RpoS-dependent sRNA RgsA regulates Fis and AcpP in Pseudomonas aeruginosa. Mol Microbiol 102:244–259. doi:10.1111/mmi.13458 27381272

[37] Koeppen K, Hampton TH, Jarek M, Scharfe M, Gerber SA, Mielcarz DW, Demers EG, Dolben EL, Hammond JH, Hogan DA, Stanton BA. 2016. A novel mechanism of host-pathogen interaction through sRNA in bacterial outer membrane vesicles. PLoS Pathog 12:e1005672. doi:10.1371/journal.ppat.1005672 27295279 PMC4905634

[38] Coleman SR, Bains M, Smith ML, Spicer V, Lao Y, Taylor PK, Mookherjee N, Hancock REW. 2021. The small RNAs PA2952.1 and PrrH as regulators of virulence, motility, and iron metabolism in Pseudomonas aeruginosa. Appl Environ Microbiol 87:e02182-20. doi:10.1128/AEM.02182-20 33158897 PMC7848907

[39] Rathinam VAK, Zhao Y, Shao F. 2019. Innate immunity to intracellular LPS. Nat Immunol 20:527–533. doi:10.1038/s41590-019-0368-3 30962589 PMC7668400

[40] Yahr TL, Wolfgang MC. 2006. Transcriptional regulation of the Pseudomonas aeruginosa type III secretion system. Mol Microbiol 62:631–640. doi:10.1111/j.1365-2958.2006.05412.x 16995895

[41] Shrestha M, Bernhards RC, Fu Y, Ryan K, Schubot FD. 2020. Backbone interactions between transcriptional activator ExsA and anti-activator ExsD facilitate regulation of the type III secretion system in Pseudomonas aeruginosa. Sci Rep 10:9881. doi:10.1038/s41598-020-66555-z 32555263 PMC7303211

[42] Park J-W, Kim Y-J, Shin I-S, Kwon O-K, Hong JM, Shin N-R, Oh S-R, Ha U-H, Kim J-H, Ahn K-S. 2016. Type III secretion system of Pseudomonas aeruginosa affects matrix metalloproteinase 12 (MMP-12) and MMP-13 expression via nuclear factor ΚB signaling in human carcinoma epithelial cells and a pneumonia mouse model. J Infect Dis 214:962–969. doi:10.1093/infdis/jiw278 27377745

[43] Wilderman PJ, Sowa NA, FitzGerald DJ, FitzGerald PC, Gottesman S, Ochsner UA, Vasil ML. 2004. Identification of tandem duplicate regulatory small RNAs in Pseudomonas aeruginosa involved in iron homeostasis. Proc Natl Acad Sci U S A 101:9792–9797. doi:10.1073/pnas.0403423101 15210934 PMC470753

