# Supplementary material for: The small RNA PrrH aggravates Pseudomonas aeruginosa-induced acute lung injury by regulating the type III secretion system activator ExsA
Source: Microbiol Spectr. 2024 Jan 30;12(3):e00626-23. doi: 10.1128/spectrum.00626-23 (PMC10913731; doi:10.1128/spectrum.00626-23)
Supplement: Supplemental material — Fig. S1 to S4; Tables S1 to S4. [file spectrum.00626-23-s0001.pdf]

SUPPLEMENTAL MATERIAL

Figure S1. The wild-type strain (WT/Vector), the *prrH* gene-deficient strain (Δ*prrH*/Vector), the *prrH*-overexpression strain (WT/PrrH), and the *prrH*-overexpression strain in the Δ*prrH* mutation (Δ*prrH*/PrrH) were constructed in previous work. The expression levels of the PrrH in different strains were analyzed using qRT-PCR. Data are shown as mean ± SEM of at least three independent experiments (*, $P < 0.05$; **, $P < 0.01$; ***, $P < 0.001$; ns, non-significant).

Figure S2. Growth curve analysis of the WT/Vector, Δ*prrH*/Vector, PrrH/Vector, and Δ*prrH*/PrrH strains. The OD_{600} values were recorded at two-hour intervals for different strains with the same initial bacterial load. Data are shown as mean ± SEM of at least three independent experiments (*, $P < 0.05$; **, $P < 0.01$; ***, $P < 0.001$; ns, non-significant).

Figure S3. The expression levels of the LPS protein in different strains were analyzed using Elisa kit. Data are shown as mean ± SEM of at least three independent experiments (*, $P < 0.05$; **, $P < 0.01$; ***, $P < 0.001$; ns, non-significant).

Figure S4. The expression levels of the LPS-related genes in different strains were analyzed using qRT-PCR. Data are shown as mean ± SEM of at least three independent experiments (*, $P < 0.05$; **, $P < 0.01$; ***, $P < 0.001$; ns, non-significant).

Figure S1

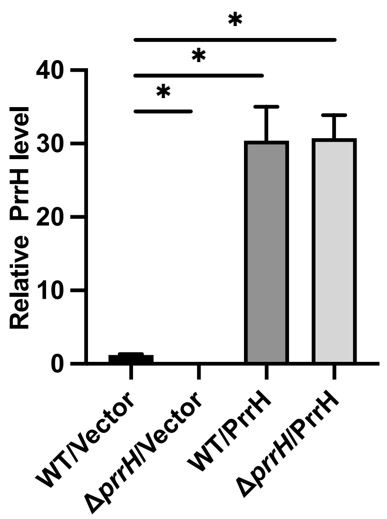

Figure S2

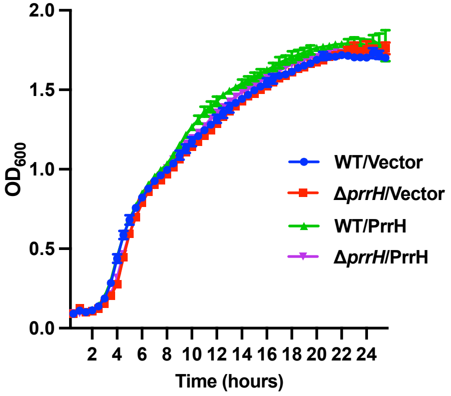

Figure S3

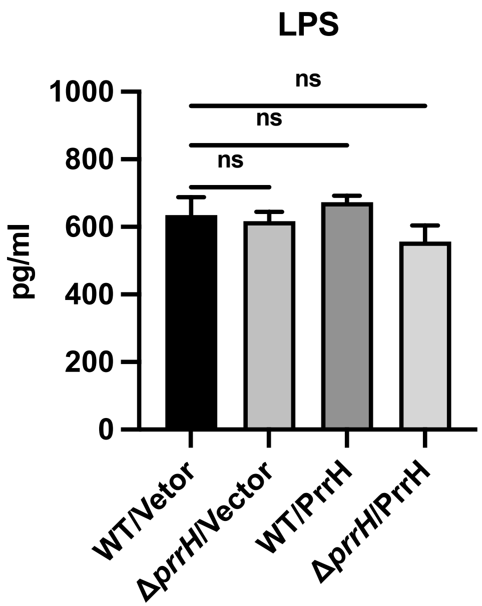

Figure S4

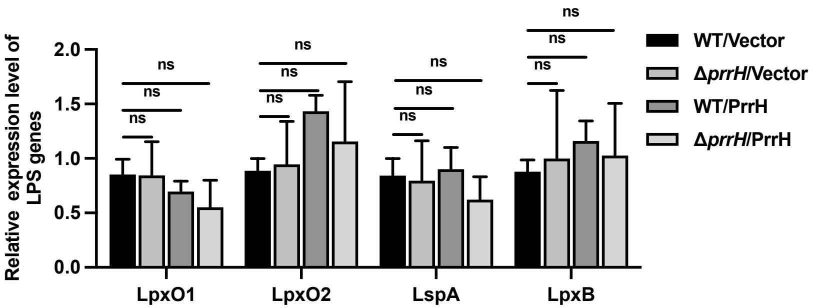

Table S1 Prediction analysis for potential PrrH targets based on data in the IntaRNA database

Target gene ID	Location in PAO1	Gene length	Product function	Binding Type
popB (PA1708)	1853665..1854837	1173	The translocation apparatus	CDS
popD (PA1709)	1854849..1855736	888	The translocation apparatus	CDS
pcrV (PA1706)	1852288..1853172	885	The translocation apparatus	CDS
exsB (PA1712)	1856562..1856975	414	The basal body	CDS
pscC (PA1716)	1859493..1861295	1803	The basal body	CDS
pcrD (PA1703)	1849403..1851523	2121	The basal body	CDS
pscI (PA1722)	1863799..1864137	339	The basal body	CDS
pscJ (PA1723)	1864134..1864880	747	The basal body	CDS
pscF (PA1719)	1862764..1863021	258	The needle filament	CDS
pscL (PA1725)	1865494..1866138	645	The needle filament	CDS
exoS (PA3841)	4303141..4304502	1362	The effector proteins	CDS
exoY (PA2191)	2410344..2411480	1137	The effector proteins	CDS
exoT (PA0044)	58786..60159	1374	The effector proteins	CDS
exsA (PA1713)	1857273..1858109	837	The regulation system	CDS
exsD (PA1714)	1858207..1859037	831	The regulation system	CDS
exsC (PA1710)	1855862..1856299	438	The regulation system	CDS
popN (PA1698)	1847227..1848093	867	The regulation system	CDS
pcrG (PA1705)	1851982..1852278	297	The regulation system	CDS
pscP (PA1695)	1844144..1845253	1110	The regulation system	CDS
pcrH (PA1707)	1853181..1853684	504	The chaperones	CDS
pscB (PA1715)	1859071..1859493	423	The chaperones	CDS
pscE (PA1718)	1862558..1862761	204	The chaperones	CDS

pscG (PA1720)	1863024..1863371	348	The chaperones	CDS
pscR (PA1693)	1842568..1843221	654	The chaperones	CDS

Table S2 Bacterial strains and plasmids

Strains	Genotype or characteristics	Source
Pseudomonas aeruginosa		
PAO1	Wild-type strain, Gm^S， Amp^R	Our lab(1)
PAO1 *ΔprrH*	Deficiency of *prrH*, Gm^S， Amp^R	Our lab
PAO1 PrrH	*prrH* expression strain, Gm^S， Amp^R	Our lab
PAO1 *ΔprrH/*PrrH	The prrH overexpression in *ΔprrH* strain, Gm^S, Amp^R	Our lab
PAO1 *ΔexsA*	Deficiency of *exsA*, Gm^S, Amp^R	This work
PAO1 *ΔexsA/*PrrH	The prrH overexpression in *ΔexsA* strain, Gm^S, Amp^R	This work
Escherichia coli		
DH5α	Wild-type strain, Gm^S, Amp^S	Our lab
SM10λπ	Wild-type strain, Gm^S, Amp^S	Our lab
Plasmids		
pGSM	Suicide plasmids to construct *ΔexsA* strains	Our lab
pQF50	Promter-less *lac*Z reporter plasmid, Amp^R	Our lab
pQF50-P*exsA*	pQF50 derivative, *lac*Z reporter plasmid, controlled by the constitutive *exsA* promoter, Amp^R	This work
pROp200	Control plasmid based on pBBR1 MCS-5, Gm^R	Our lab
pROp200-*prrH*	pROp200 derivative, *P. aeruginosa* PAO1 *prrH* overexpression plasmid, controlled by the constitutive P_{tac} promoter, Gm^R	Our lab
pSTV28	Control plasmid; containing P_{lac} promoter, Cm^R	Our lab
pSTV28-*prrH*	pSTV28 derivative, *P. aeruginosa* PAO1 *prrH* overexpression plasmid, controlled by the constitutive P_{lac} promoter, Cm^R	Our lab
pUCP32T	pucp24T derivative, P_{lac} promoter, Gm^R	Our lab

pUCP32T-*gfp*	pUCP30T and pET28-*gfp* derivative; Control plasmid; *gfp* reporter plasmid; *gfp* controlled by the constitutive P_{lac} promoter, Gm^R	Our lab
pUCP32T-*exsA-gfp*	pUCP32T-*gfp* derivative; containing transcription fusion of *exsA-gfp*; *exsA-gfp* controlled by the constitutive P_{lac} promoter, Gm^R	This work

Cm^R, Gm^R and Amp^R stand for chloramphenicol, gentamycin, and ampicillin resistance, respectively.

Table S3 Sequences of RNA and DNA oligonucleotides

Number	Primers	Primer sequence (5'→3')	Use for
**Primers for gene cloning**			
1	*exsA*-P1	ACGGCCAGTGAATTCGAGCTCACTGACCCTCGAATGCTTCG	PAO1 *ΔexsA* flanking fragement A
2	*exsA*-P2	GGCTTTCAAAAAACGATTATAAGAACCCCAACACT	PAO1 *ΔexsA* flanking fragement A
3	*exsA*-P3	TGGGGTTCTTATAATCGTTTTTTGAAAGCCCGGTA	PAO1 *ΔexsA* flanking fragement B
4	*exsA*-P4	GGCTGGATCCCAAGCTCTAGACCGTCCGATCGGCAACAAA	PAO1 *ΔexsA* flanking fragement B
5	*exsA*-M1	GCAGGTGAGGTGCTGGATG	Verify whether exsA gene is knocked out
6	*exsA*-M2	GCAATTTGGGCCGATTCTAC	Verify whether exsA gene is knocked out
**Primers for target-*gfp* translational fusion vectors**			
7	32T-exsA-5'F	GGAAACAGCTATGACTCTAGACAACAGCGACATGAGCATCG	pUCP32T-*exsA-gfp*
8	32T-exsA-3'R	CCTCTCCCTTGCTCACCATGGCCCGGCATTCGTCCTTCC	pUCP32T-*exsA-gfp*
**Primers for qPCR**			
9	RT-rpoD-5'F	CTGAAGATCGCCAAAGAGCC	qRT-PCR
10	RT-rpoD-3'R	GTGTGGTCGGTGTTCATGTC	qRT-PCR

11	RT-exoS-5’F	GCTGATCGACCAAGGTAT	qRT-PCR
12	RT-exoS-3’R	GGGTGCCACGGAAAGT	qRT-PCR
13	RT-exoT-5’F	GAGGCGGTGAAAGAGGG	qRT-PCR
14	RT-exoT-3’R	GCCGAACAGGGTGGTTAT	qRT-PCR
15	RT-exoY-5’F	TCTATGGCAGGGAGGAT	qRT-PCR
16	RT-exoY-3’R	CGTCGCTGTGGTGAAA	qRT-PCR
17	RT-pcrV-5’F	GGTCGGCAATTTCGC	qRT-PCR
18	RT-pcrV-3’R	GTGTCGTTGAGCAGGGT	qRT-PCR
19	RT-popB-5’F	CTGGGCGGCAAGTT	qRT-PCR
20	RT-popB-3’R	TGGAATCCCGAATGAG	qRT-PCR
21	RT-popD-5’F	GATGGGCGCAACGAA	qRT-PCR
22	RT-popD-3’R	TCTGGACGAAGGACTGGA	qRT-PCR
23	RT-exsA-5’F	TCAGTCCTATTTCACCCAG	qRT-PCR
24	RT-exsA-3’R	GGCATTCGTCCTTCC	qRT-PCR
25	RT-pscL-5’F	GGCACTGGCATTGGT	qRT-PCR
26	RT-pscL-3’R	GAAATCCTTGAGCACCC	qRT-PCR
27	RT-LpxO1-5’F	ATTGAGCGACCACTCCAGC	qRT-PCR
28	RT-LpxO1-3’R	TGCGGTGCCAGGTAAGG	qRT-PCR
29	RT-LpxO2-5’F	ATGTTCACCCTGCTTCCC	qRT-PCR
30	RT-LpxO2-3’R	CTTCGCCATCACGCCAC	qRT-PCR
31	RT-LspA-5’F	TGGTGGTCTGGCTGAAA	qRT-PCR
32	RT-LspA-3’R	GCGGTCGTAGAGGTTGC	qRT-PCR
33	RT-LpxB-5’F	GGTGGTGGCTTATCGGGTCG	qRT-PCR
34	RT-LpxB-3’R	CAACAGGTTCGGCAGGGAG	qRT-PCR

1. Lu Y, Li H, Pu J, Xiao Q, Zhao C, Cai Y, Liu Y, Wang L, Li Y, Huang B, Zeng J, Chen C. 2019. Identification of a novel RhlI/R-PrrH-LasI/Phzc/PhzD signalling cascade and its implication in P. aeruginosa virulence. Emerg Microbes Infect 8:1658-1667.

Table S4 GFP reporter system-related gene sequences

Name	sequence
Predicted binding sequence of *prrH*	5'-cggctggcgatggaatgaatgaga-3'
Sequences with mutations in the PrrH binding site	5'-gccgaccgctaccttacttactct-3'
Predicted binding sequence of *exsA*	5'-tttcacccagagctatcgccgccg-3'
Sequence after mutation in the ExsA binding site	5'-aagtccgtgaggatagcggcggc-3'
